# Association of alcohol control policies with adolescent alcohol consumption and with social inequality in adolescent alcohol consumption: A multilevel study in 33 countries and regions

**DOI:** 10.1016/j.drugpo.2020.102854

**Published:** 2020-10

**Authors:** E. Leal-López, C. Moreno-Maldonado, J. Inchley, B. Deforche, T. Van Havere, J. Van Damme, T. Buijs, I. Sánchez-Queija, D. Currie, A. Vieno, B. De Clercq

**Affiliations:** a Department of Developmental and Educational Psychology. University of Seville. C/Camilo José Cela, s/n. 41018, Sevilla, Spain; b MRC/CSO Social and Public Health Sciences Unit, University of Glasgow. 200 Renfield St, Glasgow G2 3AX, UK; c School of Medicine, University of St Andrews. North Haugh, St Andrews, KY16 9TF, UK; d Department of Public Health and Primary Care, Ghent University. Academical Hospital, 4K3. Corneel Heymanslaan, 10, B-9000 Ghent, Belgium; e Physical Activity, Nutrition and Health Research Unit, Faculty of Physical Education and Physical Therapy, Vrije Universiteit Brussel, Pleinlaan 2, 1050, Brussels, Belgium; f School of Social Welfare, University of Applied Sciences of Ghent. Hoogpoort 15, 9000, Ghent, Belgium; g Flemish Expertise Centre on Alcohol and other Drugs, Vanderlindenstraat 15, 1030, Brussels, Belgium; h Department of Developmental and Social Psychology, University of Padova. Via Venezia, 8 35131 – Padova, Italy

**Keywords:** Drinking, Policy, Social inequality, Young people, Cross-national, Trends

## Abstract

***Background*** Previous research found inconsistent associations between alcohol control policies and socioeconomic inequality with adolescent drinking outcomes. This study expands the focus beyond individual associations to examine whether a combination of policies is related to socioeconomic inequality in adolescent drinking outcomes and whether this relationship varies across survey years.

***Methods*** Multilevel modelling of 4 waves of repeat cross-sectional survey data (2001/02, 2005/06, 2009/10, and 2013/14) from the Health Behaviour in School-aged Children (HBSC) study was carried out. The sample was composed of 671,084 adolescents (51% girls) aged 11, 13, and 15 (mean age=13.58; SD=1.65) from 33 European and North American countries/regions. The dependent variables were lifetime alcohol consumption, weekly alcohol consumption, and lifetime drunkenness. Independent variables were of three types: individual-level variables (age, sex, Family Affluence Scale, and the Perceived Family Wealth), time-level variable (survey year), and context-level variables (minimum legal drinking age, physical availability, advertising restrictions, a total alcohol policy index, and affordability of alcohol).

***Results*** The total alcohol policy index showed a negative relationship with both lifetime and weekly consumption. Higher affordability of alcohol was related to higher lifetime and weekly consumption and higher lifetime drunkenness. Family Affluence Scale was positively related to all three alcohol measures and Perceived Family Wealth was negatively related to lifetime drunkenness, with these associations increasing across survey years. The total alcohol policy index buffered the associations of Family Affluence Scale and Perceived Family Wealth with adolescent drinking outcomes.

***Conclusion*** A combination of alcohol control policies is more effective in reducing adolescent drinking outcomes than single policy measures. Reducing the affordability of alcohol stood out as the most successful single measure. Socioeconomic inequalities (i.e. higher alcohol consumption and drunkenness in adolescents with higher family affluence and higher drunkenness in adolescents perceiving their families to be poor) have persisted and even increased across survey years. A combined alcohol control policy can help in tackling them.

## Highlights

• A combined alcohol control policy showed to be effective in reducing teen drinking.
• The combination can also tackle socioeconomic inequalities present in teen drinking.
• Single measures were in general not associated with decreases in teen drinking.
• Alcohol pricing policy showed to be the most successful single measure.

The harmful use of alcohol is one of the most important risk factors for population health worldwide, causing more than 200 disease and injury conditions, and being responsible for 3 million deaths every year (5.3% of all deaths) (World Health Organization, 2018). Special attention needs to be paid to adolescent drinking. First, consuming alcohol in adolescence has been shown to be related to significant differences in brain structure and functioning (Feldstein-Ewing, Sakhardande & Blakemore, 2014) as well as to different physical and mental health problems, and other risk behaviours such as delinquency and sexual risk-taking behaviour (Lavikainen, Salmi, Aaltonen & Lintonen, 2011; Newbury-Birch et al., 2009). Second, an association between early initiation and alcohol use disorders in adulthood has been found (Waller, Murray, Shaw, Forbes & Hyde, 2018).

Marked decreases in adolescent alcohol consumption have been observed across many countries in recent years, including Europe (Inchley et al., 2018, with HBSC data) and the USA (Miech et al., 2018). However, prevalence still remains higher than desired owing to its adverse impact on adolescent development and future health. Given the severity of the situation, a decrease of 10% in the volume of alcohol use by 2025 was established by the World Health Organization (WHO) as one of nine voluntary targets for non-communicable diseases. However, there is no international public health treaty on alcohol, and policy initiatives are recommended only in general terms. In an attempt to address the problem, a number of policy measures have been implemented by national governments. These policy initiatives can be divided into three major groups: restricting alcohol availability, regulating alcohol advertising, and controlling alcohol pricing.

The most commonly used measure to restrict alcohol availability is to impose a minimum legal drinking age (MLDA). Evidence suggests this can have a positive impact on public health outcomes such as a decrease in alcohol-related traffic accidents (Wagenaar & Toomey, 2002) and reduced mortality and morbidity rates in young people (Zhang & Caine, 2011). However, mixed results have been found regarding alcohol consumption. While most studies concluded that MLDA was related to decreases in adolescent drinking (Subbaraman & Kerr, 2013; Wagenaar & Toomey, 2002), some reported only a temporary effect (Miron & Tetelbaum, 2009) or an impact on only a specific drinking behavior such as binge drinking (Plunk, Cavazaos-Rehg, Bierut & Grucza, 2013). Other policies targeting alcohol availability include restrictions on outlet density, retail monopoly, and the hours and days of alcohol sales. These three measures are usually cited as being effective at reducing alcohol consumption and related harms (Burton et al., 2017; Holm, Veerman, Cobiac, Ekholm & Diderichsen, 2014; World Health Organization, 2018). The second group of initiatives includes policies regulating alcohol advertising. Some studies indicate that these are an effective way of reducing alcohol consumption (Holm et al., 2014), while others found a lack of robust evidence for or against such measures (Siegfried et al., 2014). The third group of initiatives refers to policies controlling the price of alcohol. An overall negative relationship has been observed between price and alcohol consumption (Wagenaar, Salois & Komro, 2009) although mixed results have been found regarding adolescent alcohol use, especially binge drinking (Nelson, 2015).

Inequalities in alcohol consumption should be considered when developing policy interventions. Alcohol consumption is typically influenced by socioeconomic status (SES) in the sense that rates of drinking are related to higher income, both at an individual and at a population level (Collins, 2016; World Health Organization, 2018). However, with respect to adolescent drinking, evidence is inconsistent. Possible explanations are that the association is dependent on the alcohol measure used or that it differs according to the indicator employed to assess socioeconomic position. For this reason, we use three different measures of adolescent drinking and two different measures of socioeconomic status to provide a more comprehensive analyses of inequalities in adolescent alcohol use. The family affluence scale (FAS) is one of the most commonly used indicators of socioeconomic status among adolescents. This scale is used to evaluate material assets within the home (e.g., the number of cars and computers). Results on the association between FAS and alcohol consumption are mixed, with some studies showing higher alcohol use related to higher FAS, others to lower FAS, while other studies concluded that there was no association (Hanson & Chen, 2007). Furthermore, there is growing evidence suggesting that relative deprivation –measured by indicators such as perceived family wealth (PFW)– is strongly related to adolescent health and lifestyles, even after taking into account the effect of other socioeconomic indicators (Goodman, Huang, Schafer-Kalkhoff & Adler, 2007). In line with FAS, results are inconsistent. Some studies found that a higher PFW was a protective factor for alcohol consumption (Liu et al., 2018), whereas others found the opposite (Zaborskis, Sumskas, Maser & Pudule, 2006).

Finally, differences have also been reported in relation to socioeconomic trends in alcohol use. Whereas some studies have reported an overall decrease in adolescent drinking in all SES groups – for example, in Australia (Livingston, 2014), Germany (Richter, Kuntsche, de Looze & Pfoertner, 2013), and the United States (Twenge & Park, 2017) – others found that the decrease was not the same for all SES groups, with higher levels of drinking being maintained among adolescents from lower SES groups, for example, in Finland (Liu et al., 2018) and New Zealand (Jackson et al., 2017). Policies and interventions aimed at promoting healthy habits and reducing risk behaviours such as alcohol consumption might have different effect on adolescents from different socioeconomic backgrounds, and interventions can narrow, widen, or have no effect in the existing socioeconomic inequalities (Moore, McDonald, Carlon & O'Rourke, 2015). In fact, there is a concern about universal public health interventions having the potential to increase social inequality in the population (Babones, 2009). To the best of our knowledge, there is no international study investigating the association between alcohol control policies and social inequality and trends in social inequality in adolescent alcohol consumption.

The present study aims to analyze the association between (i) alcohol control policies and adolescent drinking outcomes (ii) socioeconomic inequality and adolescent drinking outcomes and (iii) a combination of policies and trends in socioeconomic inequality in adolescent drinking outcomes.

## Methods

### Participants

The study sample comprised 671,084 adolescents (51% girls) aged 11, 13, and 15 (mean age=13.58; SD=1.65) from the 33 European and North American countries and regions which participated in the 2001/02, 2005/06, 2009/10, and 2013/14 surveys of the Health Behaviour in School-aged Children (HBSC) study. The HBSC study is a WHO collaborative cross-national study conducted every four years to investigate health, health-related behaviours, and social contexts of adolescents in a growing number of countries in Europe and North America.

### Procedure

Data collection was carried out through a school-based survey using classroom self-administered questionnaires. Each participant country followed a standardized international research protocol. All procedures were in accordance with the ethical standards of the institutional and/or national research committee of every country and with the 1964 Helsinki declaration and its later amendments or comparable ethical standards. Further information about the study can be found elsewhere (Roberts et al., 2007).

### Measures

Detailed information on the measures used in the present study are shown in Table 1. The data consisted of three types of variables: individual-level variables, a time-level variable, and context-level variables. At the individual level, both dependent and independent variables can be distinguished. Dependent variables were: (i) ‘Lifetime alcohol consumption’; (ii) ‘Weekly alcohol consumption’; and (iii) ‘Lifetime drunkenness’. Independent variables were age, sex, and to measure adolescents’ socioeconomic position, Family Affluence (FAS) and Perceived Family Wealth (PFW). Given that FAS and PFW show low correlations and seem to represent different constructs (Elgar et al., 2016; Moreno-Maldonado, Moreno, Ramos & Rivera, 2018), both indicators were included in this study.

**Table 1:** Measures used in the present study.

**Dependent variables**	
Lifetime alcohol consumption	‘On how many days (if any) have you drunk alcohol?’ Answer categories were: *‘Never’, ‘1–2 days’, ‘3–5 days’, ‘6–9 days’, ‘10–19 days’, ‘20–29 days’,* and *‘30 days (or more)’*. Responses were coded as abstinence (0) and at least once (1).
Weekly alcohol consumption	Students were asked if they had ever consumed alcohol and if so, how often they consumed different types of alcoholic beverages. Beverages included were beer, wine, spirits, alcopops, aperitifs, cider, cocktail and other. Answer categories were: *‘never’, ‘exceptionally’, ‘monthly’, ‘weekly’* and *‘daily’* alcohol consumption regardless the type of beverage. Responses were coded as less than weekly (0) and weekly or more frequently (1).
Lifetime drunkenness	‘Have you ever had so much alcohol that you were really drunk?’ Answer categories were: ‘*No, never’, ‘Yes, once’, ‘Yes, 2–3 times’, ‘Yes, 4–10 times’,* and *‘Yes, more than 10 times’.* Responses were coded as never or once (0) and two or more (1).
**Independent variables**	
Sociodemographic variables	Sex (*boy* and *girl*) and age (*11, 13*, and *15*).
Year of data collection	*2002, 2006, 2010*, and *2014*.
Socioeconomic status (SES)	The Family Affluence Scale (FAS) was composed of four items until 2012 (Currie et al., 2008): ‘During the past 12 months, how many times did you travel away on holiday with your family?’ (0 = *not at all*, 1 = *once*, 2 = *twice*, 3 = *more than twice*); ‘Do you have your own bedroom for yourself?’ (0 = *no*; 1 = *yes*); ‘How many computers does your family own?’ (0 = *none*, 1 = *one*, 2 = *two*, 3 = *more than two*); ‘Does your family own a car, van or truck?’ (0 = *no*; 1 = *yes one*; 2 = *yes two or more*). From 2012 onwards, two new items were added: ‘How many bathrooms does your house have?’ (1= *none*, 2 = *one,* 3 = *two*, 4 = *more than two*) and ‘Do you have a dishwasher at home?’ (1 = *no*, 2 = *yes*). Responses to all items were summed and ranked from low to high. Higher scores indicated greater family affluence. Then, in order to create a meaningful hierarchy of material wealth, all values were transformed to country- and time-specific ridit scores, which have a consistent normal distribution and a range from 0 to 1 (Donaldson, 1998).
	Perceived Family Wealth (PFW) was measured by asking the following question ‘How well-off is your family compared to others?’ Answer categories were: 1=‘*not at all well-off*’, 2=‘*not very well off*’, 3=‘*average*’, 4=‘*quite well-off*’ and 5=‘*very well-off*’.
Minimum drinking legal Age (MLDA)	The MLDA was used with two categories: 0=*’Lower than 18′* and 1= *‘18 years old’*
Physical availability	The ‘Availability Index’ evaluates the stringency of national policies implemented to restrict the access to alcohol. It was based on the index used by Brand, Saisana, Rynn, Pennoni and Lowenfels (2007) and comprised of four measures: - National minimum legal drinking age (MLDA) (3 stars): ‘*no*’ and ‘*yes’*. - Government monopoly (2 stars): ‘*no’*. ‘*partial’*, and ‘*full’*. - Outlet density restriction (2 stars): ‘*no’*, ‘*wine only*’, ‘*wine and spirits*’, and ‘*all beverages’*. - Restrictions on sales time (2 stars): ‘*none’*, ‘*on hours or days*’, and ‘*on both hours and days*’. Each measure was rated with stars according to their effectiveness (as shown by previous research). Higher ratings corresponded with higher weight in the overall index (3-star: 7.9 points, 2-star: 5.3 points, 1-star: 2.6 points). Categories were awarded proportionate scores, with the least stringent category earning no points and the highest stringency category earning full points. The ‘Availability Index’ has a range from 0 to 23.80.
Alcohol advertising restrictions	The advertising Index was based on the Brand et al. (2007) but adapted according to the method proposed by Carragher, Byrnes, Doran and Shakeshaft (2014). Media included in this study were: print, broadcast, billboards, as well as sport sponsorship and the internet. Within each category, proportionate points were given based on whether there was a complete ban, partial statutory restrictions, a voluntary self-regulated code or no restrictions at all. Moreover, within each media, regulatory differences between types of alcoholic beverage were accounted for. The ‘Advertising Index’ has a range from 0 to 2.60.
Alcohol affordability	The ‘Affordability Index’ was added in accordance to Rabinovich et al. (Rabinovich et al., 2009). This measure represents an index of gross disposable household income per capita over an index of relative alcohol price (i.e. alcohol price index over general consumer price index). Disposable household income is a measure representing the amount of money households retain for spending and saving after income taxes have been accounted for. Adjusted disposable income gives a broader picture by including social transfers that households receive free of charge from the government and/or other institutions (Eurostat. Eurostat datbase 2016)). Note that all indices were constructed with the base year set at 2015. Equations for each variable were: Alcohol price index: $\mathrm{Inde}x_{\mathrm{year},\;\mathrm{country}}=\frac{\mathrm{Alcohol}\;\mathrm{pric}e_{\mathrm{year},\;\mathrm{country}}}{\mathrm{Alcohol}\;\mathrm{pric}e_{2015,\;\mathrm{country}}}\;x\;100$ Relative alcohol price index: $\text{Inde}x_{\text{year},\;\text{country}}=\frac{\frac{\text{Alcohol}\;\text{pric}e_{\text{year},\;\text{country}}}{\text{Alcohol}\;\text{pric}e_{2015,\;\text{country}}}}{\frac{\text{General}\;\text{consumer}\;\text{price}\;\text{inde}x_{\text{year},\;\text{country}}}{\text{General}\;\text{consumer}\;\text{price}\;\text{inde}x_{2015,\;\text{country}}}}x\;100$ Gross disposable income index: $\text{Inde}x_{\text{year},\;\text{country}}=\frac{\text{Gross}\;\text{disposable}\;\text{income}\;\text{per}\;\text{capit}a_{\text{year},\;\text{country}}}{\text{Gross}\;\text{disposable}\;\text{income}\;\text{per}\;\text{capit}a_{2015,\;\text{country}}}\;x\;100$ Alcohol affordability index: $\text{Inde}x_{\text{year},\;\text{country}}=\frac{\text{Gross}\;\text{disposable}\;\text{income}\;\text{inde}x_{\text{year},\;\text{country}}}{\text{Relative}\;\text{alcohol}\;\text{price}\;\text{inde}x_{\text{year},\;\text{country}}}$ The ‘Affordability Index’ has a range from −0.34 to 1.04.
Total alcohol policy Index (Total-API)	The Total-API was computed as the sum of the Availability Index and the Advertising Index. The Total-API index has a range from 0 to 26.11.
Sources of data collection about national alcohol control policies	Cross-referencing published reports (Amphora Project, 2010; World Health Organization, 2018; Mulder and de Greeff, 2013), the WHO's Global Information System on Alcohol and Health (WHO, 2016a), the European center for Monitoring Alcohol Marketing (European Centre for Monitoring Alcohol Marketing, 2016), and the Alcohol Policy Timeline Database (WHO, 2016b). Information regarding demographics, harmonized indices of consumer prices, and price level indicators were collected through the Statistical Office of the European Union (Eurostat. Eurostat datbase 2016), except for Canada, Israel, North Macedonia, Russia, and Ukraine which were collected through the website of their respective national bureau of statistics because they were not present in Eurostat (CBS, 2016; MAKStat, 2016; Rosstat, 2016; Statcan, 2016; Ukrstat, 2016).

The time-level variable was the year of data collection (2002, 2006, 2010, and 2014). On the context-level we can distinguish between school- and country-level. School-level variables were not included but the model accounts for school-level clustering in standard errors and variance partitioning, i.e. we take into account the school level variance via the random intercept model, but no explanatory independent variables were introduced at the school-level because we did not aim to distinguish the effect of school characteristics within this study. At country-level, physical availability, advertising restrictions, and affordability of alcohol were added. In addition, minimum legal drinking age (MLDA) was also examined as this is the most popular measure in the physical availability category. In order to assess the effect of a combination of alcohol control policy initiatives, a total alcohol policy index (Total-API), as the sum of availability and advertising, was included. Data on individual-level variables and time-level variable were collected from HBSC study. Context-level information was collected from different sources (see Table 1).

### Data analyses

Multilevel modelling was performed using MLwiN 2.32 software. Four-level hierarchical models were estimated including students (level 1), school (level 2), country-years (level 3), and countries (level 4). A stepwise approach was followed to investigate the study aims. In order to confirm the four-level structure of the data, we first estimated an intercept-only model. Then, sociodemographic variables (age and sex) were incorporated (model 1). In order to evaluate whether national level alcohol control policies were associated with changes in adolescent alcohol consumption (aim i), the time variable (model 2), MLDA (model 3), the Availability Index (model 4a), the Advertising Index (model 4b), Total-API (model 4c), and the Affordability Index (model 5), were sequentially included. Note that MLDA, Availability and Advertising closely reflect policy decisions, whereas Affordability is a pricing index that is mainly an economic result of market processes. However, pricing policy as an instrument of alcohol control policies can have a potential impact on alcohol consumption via Affordability. Therefore, Affordability was not included in the Total-API as this variable measures the market price and not the pricing policy of a specific country.

To analyse socioeconomic inequality in adolescent drinking outcomes (aim ii), FAS (model 6a), PFW (model 6b), and both FAS and PFW simultaneously (model 6c) were incorporated to test whether SES was related to adolescent alcohol drinking outcomes. Finally, to test the third aim, the interaction between Total-API and SES (model 7a), the interaction between time and SES (model 7b), and the interaction between time, SES, and Total-API (model 7c) were added to examine whether this combined alcohol control policy index was related to trends in socioeconomic inequality in adolescent alcohol consumption. In the last three models, SES corresponded to FAS, PWF, or both considering whether they were significant or not in models 6a and 6b. All models were estimated using the maximum likelihood procedure, using the (Restricted) Iterative Generalized Least Squares algorithm. The variance partition coefficient (VPC) indicates the proportion of variance in a measure of alcohol that is attributable to differences between specific analytical levels (e.g. schools) (Snijders & Bosker, 2012).

## Results

Descriptive statistics of the sample are shown in Table 2.

**Table 2:** Descriptive statistics of the characteristics of the sample.

			Survey year								TOTAL	
			2002		2006		2010		2014			
			N (%)	Mean (SD)	N (%)	Mean (SD)	N (%)	Mean (SD)	N (%)	Mean (SD)	N (%)	Mean (SD)
Sex		Boy	75,709 (48.8)		82,205 (49.0)		84,803 (49.1)		86,242 (49.2)		328,959 (49.0)	
		Girl	79,591 (51.2)		85,451 (51.0)		87,926 (50.9)		89,157 (50.8)		342,125 (51.0)	
		Missing	0 (0)		0 (0)		0 (0)		0 (0)		0 (0)	
Age category		11	52,773 (34.3)		53,433 (32.1)		55,477 (32.4)		56,048 (32.2)		217,731 (32.7)	
		13	52,937 (34.4)		56,445 (33.9)		57,532 (33.6)		60,333 (34.6)		227,247 (33.9)	
		15	48,296 (31.4)		56,631 (34.0)		58,107 (34.0)		57,771 (33.2)		220,805 (32.9)	
		Missing	1294 (0.8)		1147 (0.7)		1613 (0.9)		1247 (0.7)		5304 (0.8)	
Family Affluence Scale (FAS)				0.41 (0.273)		0.47 (0.281)		0.56 (0.281)		0.55 (0.279)		0.50 (0.285)
Missing			3494 (2.3)		6008 (3.7)		8351 (5.1)		13,533 (8.4)		31,386 (4.7)	
Perceived Family Wealth (PFW)				3.58 (0.880)		3.65 (0.892)		3.63 (0.895)		3.59 (0.890)		3.61 (0.890)
Missing			2517 (1.6)		4249 (2.5)		6379 (3.7)		7668 (4.4)		20,813 (3.1)	
Lifetime alcohol consumption	Never		110,183 (70.9)		111,512 (66.5)		126,282 (73.1)		139,430 (79.5)		487,407 (72.6)	
	At least once		35,026 (22.6)		41,838 (25.0)		38,640 (22.4)		24,648 (14.1)		140,152 (20.9)	
	Missing		10,091 (6.5)		14,306 (8.5)		7807 (4.5)		11,321 (6.5)		43,528 (6.5)	
Frequency of alcohol consumption	Less than weekly		126,080 (81.2)		131,558 (78.5)		146,046 (84.6)		153,419 (87.5)		557,103 (83.0)	
	At least weekly		19,129 (12.3)		21,792 (13.0)		18,876 (10.9)		10,659 (6.1)		70,456 (10.5)	
	Missing		10,091 (6.5)		14,306 (8.5)		7807 (4.5)		11,321 (6.5)		43,528 (6.5)	
Lifetime drunkenness	Never or once		128,696 (82.9)		130,070 (77.6)		142,059 (82.2)		150,174 (85.6)		550,999 (82.1)	
	At least twice		25,367 (16.3)		25,606 (15.3)		25,016 (14.5)		16,568 (9.4)		92,557 (13.8)	
	Missing		1237 (0.8)		11,980 (7.1)		5654 (3.3)		8657 (4.9)		27,531 (4.1)	

Results of the regression analyses are presented in Table 3, Table 4, Table 5. In order to justify the four-level structure, an intercept-only model was first estimated for each outcome measure. In all cases, the model fit improved (in comparison with the single-level model) when the random intercepts were added. Regarding the VPC, results for lifetime alcohol consumption indicated that 18.63% of the total variance was at the school-level, 6.45% at the country-year level, and 5.13% at the country-level. For weekly consumption, 16.60% of the total variance was at the school-level, 6.45% at the country-year level, and 6.90% at the country-level. For lifetime drunkenness, 14.72% was at the school-level, 4.53% at the country-year level, and 6.13% at the country-level. In model 1, both age and sex were significant, revealing that older adolescents and males were more likely to have ever consumed alcohol, to drink weekly, and to have been drunk at least twice in their lives. In model 2, time showed a downward trend in all three alcohol drinking outcomes between 2002 and 2014. At this point, context-level variables related to alcohol control policy and SES variables were incorporated in the following models. Results for each outcome measure are described below.

**Table 3:** Regression results for lifetime consumption.

	**Nullmodel**		**Model 1**		**Model 2**		**Model 3**		**Model 4**						**Model 5**		**Model 6**				**Model 7**					
									**a**		**b**		**c**				**a**		**b**		**a**		**b**		**c**	
	**β**	**S.E.**	**β**	**S.E.**	**β**	**S.E.**	**β**	**S.E.**	**β**	**S.E.**	**β**	**S.E.**	**β**	**S.E.**	**β**	**S.E.**	**β**	**S.E.**	**β**	**S.E.**	**β**	**S.E.**	**β**	**S.E.**	**β**	**S.E.**
**Constant**	−1.50***	0.086	−1.43***	0.076	−1.19***	0.084	−1.29***	0.108	−1.21***	0.083	−1.23***	0.083	−1.22***	0.083	−1.30***	0.097	−1.29***	0.098	−1.30***	0.098	−1.29***	0.098	−1.29***	0.098	−1.29***	0.098
**Age**			0.61***	0.003	0.61***	0.003	0.61***	0.003	0. 0.61***	0.003	0.61***	0.003	0.61***	0.003	0.61***	0.003	0.62***	0.004	0.61***	0.004	0.62***	0.004	0.62***	0.004	0.62***	0.004
**Sex**			−0.39***	0.007	−0.39***	0.007	−0.39***	0.007	−0.39***	0.007	−0.39***	0.007	−0.39***	0.007	−0.33***	0.008	−0.33***	0.008	−0.33***	0.008	−0.33***	0.008	−0.33***	0.008	−0.33***	0.008
**Time**					−0.49***	0.074	−0.53***	0.076	−0.46***	0.077	−0.43***	0.082	−0.45***	0.078	−0.33***	0.101	−0.38***	0.104	−0.33***	0.103	−0.38***	0.104	−0.38***	0.104	−0.38***	0.104
																										
**MLDA**							0.20	0.127																		
																										
**Availability**									−0.02	0.011	−0.02	0.011														
**Advertising**											−0.11	0.096														
**Total-API**													−0.02	0.010	−0.02*	0.011	−0.02*	0.011	−0.02*	0.011	−0.02*	0.011	−0.02*	0.011	−0.02*	0.011
**Affordability**															0.89***	0.218	0.89***	0.222	0.91***	0.221	0.89***	0.222	0.89***	0.222	0.89***	0.222
																										
**FAS**																	0.48***	0.015			0.49***	0.015	0.50***	0.023	0.49***	0.024
**PFW**																			−0.003	0.005						
																										
Total-API*FAS																					−0.02***	0.002			−0.02***	0.004
Time*FAS																							−0.03	0.030	−0.004	0.030
Time*FAS*Total-API																									0.003	0.005
**Random effects**																										
	**σ**	**S.E.**	**σ**	**S.E.**	**σ**	**S.E.**	**σ**	**S.E.**	**σ**	**S.E.**	**σ**	**S.E.**	**σ**	**S.E.**	**σ**	**S.E.**	**σ**	**S.E.**	**σ**	**S.E.**	**σ**	**S.E.**	**σ**	**S.E.**	**σ**	**S.E.**
**Country**	0.178	0.061	0.127	0.048	0.145	0.047	0.154	0.049	0.132	0.044	0.122	0.042	0.129	0.043	0.111	0.042	0.108	0.042	0.108	0.042	0.107	0.042	0.107	0.042	0.107	0.042
**Country-year**	0.227	0.037	0.241	0.036	0.164	0.025	0.159	0.025	0.165	0.026	0.168	0.026	0.165	0.026	0.149	0.028	0.157	0.030	0.155	0.029	0.158	0.030	0.158	0.030	0.158	0.030
**School**	0.753	0.015	0.186	0.006	0.192	0.006	0.191	0.006	0.192	0.006	0.192	0.006	0.192	0.006	0.172	0.007	0.171	0.007	0.172	0.007	0.170	0.007	0.171	0.007	0.170	0.007
																										
**Model Evaluation**																										
**−2 Loglikelihood**	527,332		396,953		394,898		395,353		395,015		394,967		394,938		300,883		286,273		286,011		284,634		284,569		284,620	

MLDA: Minimum Legal Age Drinking; Total-API: Total Alcohol Policy Index; FAS: Family Affluence Scale; PWF: Perceived Family Wealth.

Significance codes: ‘***’ 0.001 ‘**’ 0.01 ‘*’ 0.05.

**Table 4:** Regression results for weekly alcohol consumption.

	**Null model**		**Model 1**			**Model 2**			**Model 3**			**Model 4**									**Model 5**			**Model 6**									**Model 7**								
												**a**			**b**			**c**						**a**			**b**			**c**			**a**			**b**			**c**		
	**β**	**S.E.**	**β**		**S.E.**	**β**		**S.E.**	**β**		**S.E.**	**β**		**S.E.**	**β**		**S.E.**	**β**		**S.E.**	**β**		**S.E.**	**β**		**S.E.**	**β**		**S.E.**	**β**		**S.E.**	**β**		**S.E.**	**β**		**S.E.**	**β**		**S.E.**
**Constant**	−2.44***	0.096	−2.27***		0.089	−2.00***		0.095	−2.04***		0.120	−2.05***		0.088	−2.07***		0.085	−2.05***		0.087	−2.15***		0.104	−2.15***		0.106	−2.17***		0.105	−2.15***		0.106	−2.15***		0.105	−2.15***		0.106	−2.15***		0.105
**Age**			0.54***		0.004	0.54***		0.004	0.54***		0.004	0.54***		0.004	0.54***		0.004	0.54***		0.004	0.53***		0.004	0.53***		0.004	0.53***		0.004	0.53***		0.004	0.53***		0.004	0.53***		0.004	0.53***		0.004
**Sex**			−0.59***		0.009	−0.59***		0.009	−0.60***		0.009	−0.60***		0.009	−0.60***		0.009	−0.60***		0.009	−0.55***		0.010	−0.54***		0.011	−0.54***		0.011	−0.54***		0.011	−0.54***		0.011	−0.54***		0.011	−0.54***		0.011
**Time**						−0.55***		0.071	−0.57***		0.074	−0.50***		0.075	−0.44***		0.080	−0.48***		0.076	−0.32***		0.103	−0.37***		0.106	−0.33***		0.105	−0.37***		0.106	−0.37***		0.106	−0.37***		0.106	−0.37***		0.106
																												
**MLDA**									0.08		0.135																														
																												
**Availability**												−0.03**		0.011	−0.03**		0.011																								
**Advertising**															−0.17		0.096																								
**Total-API**																		−0.03**		0.011	−0.04***		0.012	−0.04***		0.013	−0.04***		0.012	−0.04***		0.013	−0.04***		0.012	−0.04***		0.013	−0.04***		0.012
**Affordability**																					0.82***		0.227	0.82***		0.232	0.83***		0.230	0.82***		0.232	0.82***		0.231	0.82***		0.232	0.82***		0.231
																												
**FAS**																								0.35***		0.019				0.36***		0.020	0.35***		0.019	0.35***		0.029	0.33***		0.030
**PFW**																											0.03***		0.006	−0.01		0.007									
																												
Total-API*FAS																																	−0.01***		0.003				−0.01*		0.005
Time*FAS																																				0.01		0.038	0.03		0.039
Time*FAS*Total-API																																							0.001		0.007
**Random effects**																																									
	**σ**	**S.E.**	**σ**		**S.E.**	**σ**		**S.E.**	**σ**		**S.E.**	**σ**		**S.E.**	**σ**		**S.E.**	**σ**		**S.E.**	**σ**		**S.E.**	**σ**		**S.E.**	**σ**		**S.E.**	**σ**		**S.E.**	**σ**		**S.E.**	**σ**		**S.E.**	**σ**		**S.E.**
**Country**	0.244	0.077	0.196		0.065	0.219		0.065	0.228		0.066	0.164		0.051	0.137		0.045	0.156		0.049	0.154		0.052	0.152		0.053	0.151		0.052	0.152		0.053	0.151		0.053	0.152		0.053	0.151		0.053
**Country-year**	0.227	0.038	0.249		0.038	0.149		0.024	0.149		0.024	0.152		0.024	0.156		0.025	0.152		0.024	0.149		0.029	0.158		0.030	0.156		0.030	0.158		0.030	0.157		0.030	0.158		0.030	0.157		0.030
**School**	0.655	0.017	0.181		0.008	0.183		0.008	0.185		0.008	0.187		0.008	0.188		0.008	0.187		0.008	0.171		0.009	0.171		0.009	0.170		0.009	0.171		0.009	0.171		0.009	0.171		0.009	0.170		0.009
																												
**Model Evaluation**																																									
**−2 Loglikelihood**	630,912			626,278			659,175			900,718			−402,876			−266,009			−849,433			−151,657			−113,257			−105,893			−113,523			−114,552			−113,182			−114,076	

MLDA: Minimum Legal Age Drinking; Total-API: Total Alcohol Policy Index; FAS: Family Affluence Scale; PWF: Perceived Family Wealth.

Significance codes: ‘***’ 0.001 ‘**’ 0.01 ‘*’ 0.05.

**Table 5:** Regression results for lifetime drunkenness.

	**Null Model**		**Model 1**			**Model 2**			**Model 3**		**Model 4**						**Model 5**		**Model 6**						**Model 7**					
											**a**		**b**		**c**				**a**		**b**		**c**		**a**		**b**		**c**	
	**β**	**S.E.**	**β**		**S.E.**	**β**		**S.E.**	**β**	**S.E.**	**β**	**S.E.**	**β**	**S.E.**	**β**	**S.E.**	**β**	**S.E.**	**β**	**S.E.**	**β**	**S.E.**	**β**	**S.E.**	**β**	**S.E.**	**β**	**S.E.**	**β**	**S.E.**
**Constant**	−1.62***	0.089	−1.57***		0.085	−1.37***		0.089	−1.52***	0.104	−1.37***	0.091	−1.37***	0.092	−1.37***	0.091	−1.52***	0.095	−1.52***	0.096	−1.52***	0.095	−1.52***	0.095	−1.51***	0.095	−1.52***	0.095	−1.52***	0.095
**Age**			0.78***		0.005	0.78***		0.005	0.78***	0.005	0.78***	0.005	0.78***	0.005	0.78***	0.005	0.80***	0.006	0.80***	0.006	0.80***	0.006	0.80***	0.006	0.80***	0.006	0.80***	0.006	0.80***	0.006
**Sex**			−0.27***		0.008	−0.27***		0.008	−0.27***	0.008	−0.27***	0.008	−0.27***	0.008	−0.27***	0.008	−0.23***	0.010	−0.23***	0.010	−0.23***	0.010	−0.23***	0.010	−0.23***	0.010	−0.23***	0.010	−0.23***	0.010
**Time**						−0.41***		0.058	−0.45***	0.060	−0.41***	0.062	−0.40***	0.066	−0.41***	0.062	−0.23**	0.085	−0.26**	0.086	−0.23**	0.085	−0.27***	0.087	−0.27**	0.087	−0.27**	0.087	−0.27**	0.087
																												
**MLDA**									0.29***	0.114																				
																												
**Availability**											−0.002	0.010	−0.001	0.010																
**Advertising**													−0.04	0.087																
**Total-API**															−0.002	0.010	0.01	0.011	0.01	0.011	0.01	0.011	0.01	0.011	0.01	0.011	0.01	0.011	0.01	0.011
**Affordability**																	0.69***	0.191	0.69***	0.194	0.70***	0.192	0.69***	0.195	0.69***	0.194	0.69***	0.194	0.69***	0.194
																												
**FAS**																			0.31***	0.018			0.39***	0.019	0.38***	0.019	0.32***	0.030	0.32***	0.030
**PFW**																					−0.05***	0.006	−0.09***	0.006	−0.07***	0.006	−0.06***	0.011	−0.06***	0.011
																												
Total-API*FAS																									−0.003	0.003			−0.01	0.005
Total-API*PFW																									−0.01***	0.001			−0.01*	0.002
Time*FAS																											0.11**	0.039	0.11***	0.039
Time*PFW																											−0.04**	0.013	−0.01	0.014
Time*FAS*Total-API																													0.003	0.007
Time*PFW*Total-API																													−0.01***	0.002
**Random effects**																														
	**σ**	**S.E.**	**σ**		**S.E.**	**σ**		**S.E.**	**σ**	**S.E.**	**σ**	**S.E.**	**σ**	**S.E.**	**σ**	**S.E.**	**σ**	**S.E.**	**σ**	**S.E.**	**σ**	**S.E.**	**σ**	**S.E.**	**σ**	**S.E.**	**σ**	**S.E.**	**σ**	**S.E.**
**Country**	0.215	0.065	0.201		0.060	0.212		0.059	0.190	0.053	0.216	0.060	0.217	0.060	0.217	0.060	0.166	0.050	0.165	0.050	0.163	0.049	0.159	0.049	0.159	0.049	0.158	0.049	0.158	0.048
**Country-year**	0.156	0.027	0.148		0.023	0.094		0.016	0.092	0.015	0.095	0.016	0.095	0.016	0.094	0.016	0.092	0.018	0.096	0.019	0.094	0.019	0.098	0.019	0.097	0.019	0.097	0.019	0.097	0.019
**School**	0.568	0.015	0.161		0.007	0.162		0.007	0.162	0.007	0.162	0.007	0.161	0.007	0.161	0.007	0.145	0.007	0.143	0.007	0.141	0.007	0.141	0.007	0.140	0.007	0.141	0.007	0.140	0.007
**Model Evaluation**																														
**−2 Loglikelihood**	346,191			275,298			274,873		274,869		274,932		274,989		274,963		198,059		188,604		187,429		186,257		186,227		185,807		185,672	

MLDA: Minimum Legal Age Drinking; Total-API: Total Alcohol Policy Index; FAS: Family Affluence Scale; PWF: Perceived Family Wealth.

Significance codes: ‘***’ 0.001 ‘**’ 0.01 ‘*’ 0.05.

### Lifetime alcohol consumption

#### Alcohol policies and lifetime alcohol consumption

Table 3 presents regression models for lifetime alcohol consumption. In model 3, the variable included was MLDA. The estimate was not significant, showing a lack of association between the MLDA and lifetime alcohol consumption. In models 4a and 4b, the Availability Index and the Advertising Index, were sequentially added. Model 4c was performed with Total-API. Results were not significant, and the models fit decreased in comparison with the simple model with sociodemographic covariates and time. After that, the Affordability Index was incorporated in model 5. Results showed that affordability was significant and positively related to lifetime alcohol consumption (β = 0.889, *p*<.001), showing that greater affordability is associated with higher lifetime consumption. Total-API showed a significant negative relationship in model 5, after controlling for affordability (β = −0.023, *p*<.05), which means that having a combination of policies in place can reduce adolescent lifetime consumption independently of affordability.

#### Social inequality in lifetime alcohol consumption

The next two models (6a and 6b) examined SES. Whereas FAS was found to be significantly and positively related to lifetime alcohol consumption (β = 0.483, *p*<.001), meaning higher lifetime consumption among adolescents pertaining to families with higher material affluence, PFW was not significant and the model fit was worse. Therefore, model 6c, including both socioeconomic indicators, is not included in Table 3.

#### Alcohol policies and trends in social inequalities in lifetime consumption

Finally, the interactions between Total-API and FAS (model 7a), time and FAS (model 7b), and time, FAS, and Total-API (model 7c) were added. Model 7a showed a significant interaction between Total-API and FAS (β = −0.015, *p*<.001), indicating that the combination of alcohol control policies partially mitigated the detrimental effect of higher family affluence on lifetime alcohol consumption. However, model 7b and 7c yielded non-significant results, showing the absence of interaction between time and FAS, as well as, between time, FAS, and Total-API.

### Weekly alcohol consumption

#### Alcohol policies and weekly alcohol consumption

Table 4 shows regression models for weekly alcohol consumption. In Model 3, MLDA was not significantly related to weekly drinking. In contrast, the Availability Index yielded a significant result in model 4a (β = −0.031, *p*<.01), indicating that countries with stricter regulations concerning the physical availability of alcohol had a lower proportion of adolescents reporting weekly alcohol consumption. The Advertising Index (model 4b) however did not result in a better model, nor was the estimate significant. The model fit of the model 4c (Total-API) was better than the model fit of model 4a (β = −0.032, *p*<.01) and Total-API was significantly related to weekly alcohol consumption, indicating that a combination of measures targeting both availability and advertising can be effective in reducing weekly drinking. The Affordability Index (model 5) was also significantly related to weekly alcohol consumption (β = 0.822, *p*<.001) such that increased affordability was associated with a higher level of weekly alcohol consumption.

#### Social inequality in weekly alcohol consumption

In models 6a and 6b, both FAS (β = 0.351, (*p*<.001) and PFW (β = 0.027, *p*<.001) were significantly and positively related to weekly consumption. However, the combination of both, tested in model 6c, showed that only FAS was related to weekly drinking (the effect of PFW was not significant when FAS was included), revealing that adolescents with higher family affluence reported higher weekly drinking.

#### Alcohol policies and trends in social inequalities in weekly consumption

The last steps analysed the possible interactions between Total-API, FAS, and time (Model 7a, b and c). The only significant interaction was found in model 7a, between Total-API and FAS (β= −0.011, *p*<.001). In line with lifetime alcohol consumption, more stringent policies reduced the detrimental effect of higher family affluence on weekly drinking.

### Lifetime drunkenness

#### Alcohol policies and lifetime drunkenness

Table 5 presents regression models for lifetime drunkenness. In this case, MLDA (model 3) showed a significant positive association (β= 0.287, *p*<.01), with higher rates of adolescent lifetime drunkenness in countries with a higher minimum legal drinking age. The Availability Index (model 4a), the Advertising Index (model 4b), and the Total-API (model 4c) did not have a significant effect but the affordability of alcohol (model 5) was positively related to lifetime drunkenness (β = 0.688, *p*<.001), showing that higher affordability of alcohol was related to higher rates of lifetime drunkenness.

#### Social inequality in lifetime drunkenness

In models 6a and 6b, both FAS (β = 0.311, *p*<.001) and PFW (β = −0.047, *p*<.001) were found to be significant and, unlike the preceding outcome measures, both indicators remained significant when combined into a single model (model 6c) (FAS: β = 0.387, *p*<.001; PFW: β = −0.087, *p*<.001). However, the estimates of FAS and PFW were opposite such that higher lifetime drunkenness was associated with higher family affluence and with lower perceived family wealth.

#### Alcohol policies and trends in social inequalities in lifetime drunkenness

Finally, interactions between Total-API, FAS/PFW, and time were incorporated in models 7a, 7b, and 7c. Model 7a showed no relation between Total-API and FAS but a significant interaction between Total-API and PFW was found (β = −0.009, *p*<.001), what means that stricter regulations on alcohol reduced the detrimental effect of inequality (i.e. higher rates of lifetime drunkenness among adolescents who perceive their families to be poor) on lifetime drunkenness. In model 7b, interactions between time and both SES measures were observed (FAS: β = 0.106, *p*<.01; PFW: β= −0.039, *p*<.01), showing that social inequalities in adolescent drunkenness have increased across survey years. Contrary to lifetime and weekly consumption, Model 7c yielded a significant three-way interaction between time, PFW, and Total-API indicating that increasing inequalities across survey years in lifetime drunkenness were reduced in countries with a higher Total-API (β =- 0.008; *p*<.001).

A summary of the significant associations between dependent and independent variables is presented in Table 6.

**Table 6:** Summary of significant associations between dependent and independent variables.

	**Lifetime consumption**	**Weekly consumption**	**Lifetime drunkenness**
**Age**	β = 0.61, SE = 0.003***	β = 0.54, SE = 0.004***	β = 0.78, SE = 0.005***
**Sex**	β = −0.39, SE = 0.007***	β = −0.60, SE = 0.009***	β = −0.27, SE = 0.008***
**Time**	β = −0.49, SE = 0.074***	β = −0.56, SE = 0.071***	β = −0.41, SE = 0.058***
**MLDA**			β = 0.29, SE = 0.114**
**Availability**		β = −0.03, SE = 0.011**	
**Advertising**			
**Total-API**	β = −0.02, SE = 0.011*	β = −0.03, SE = 0.011**	
**Affordability**	β = 0.89, SE = 0.218***	β = 0.82, SE = 0.227***	β = 0.69, SE = 0.191***
**FAS**	β = 0.48, SE = 0.015***	β = 0.36, SE = 0.020***	β = 0.39, SE = 0.019***
**PFW**			β = −0.09, SE = 0.006***
Total-API*FAS	β = −0.02, SE = 0.002***	β = −0.01, SE = 0.003***	
Total-API*PFW			β = −0.01, SE = 0.001***
Time*FAS			β = 0.11, SE = 0.039**
Time*PFW			β = −0.04, SE = 0.001**
Time*FAS*Total-API			
Time*PFW*Total-API			β = −0.01, SE = 0.002***

MLDA: Minimum Legal Age Drinking; Total-API: Total Alcohol Policy Index; FAS: Family Affluence Scale; PWF: Perceived Family Wealth.

Significance codes: ‘***’ 0.001 ‘**’ 0.01 ‘*’ 0.05.

## Discussion

The present study aimed to investigate the relationship between national alcohol control policies and (socioeconomic inequality in) alcohol consumption among adolescents aged 11–15 in 33 countries and regions across Europe and North America between 2002 and 2014. We explored associations between (i) alcohol control policies and adolescent drinking outcomes (ii) socioeconomic inequality and adolescent drinking outcomes and (iii) a combination of policies and trends in socioeconomic inequality in adolescent drinking outcomes.

Firstly, we found that a combination of policy measures (i.e. restricting alcohol availability in combination with regulating alcohol advertising) were associated with lower lifetime and weekly alcohol consumption. In addition, a decrease in affordability was related to a reduction in all three drinking outcomes. Similar results have been found in previous research (Burton et al., 2017; Meier et al., 2016; Wagenaar, Salois and Komro, 2009). It should be noted that the affordability of alcoholic beverages may decrease because they become more expensive (e.g. increased price, additional taxes) or due to a smaller budget (e.g. economic crisis, less pocket money). The latter has previously been shown to be related to reduced alcohol consumption in adolescents (Kokkevi, Stavrou, Kanavou, Fotiou & Richardson, 2018; Obradors-Rial, Ariza, Rajmil & Muntaner, 2018). On the contrary, other single policy measures such as imposing a minimum legal drinking age, restricting alcohol availability, or regulating alcohol advertising, were in general not related to adolescent alcohol consumption in the present study. The exceptions were: the restriction of physical availability seems to reduce weekly consumption and a higher minimum legal drinking age was associated with higher lifetime drunkenness, although a reversed causality is possible here (i.e. that countries which have a higher proportion of lifetime drunkenness set stricter MLDA's in an attempt to tackle the problem).

Concerning the second objective, our findings showed that living in families with higher material affluence represented a risk factor for both lifetime and weekly alcohol consumption and having been drunk. This is in line with previous studies showing that adolescents belonging to families with higher material affluence tend to report a higher alcohol consumption (Richter et al., 2013) and drunkenness (Gomes de Matos, Kraus, Hannemann, Soellner & Piontek, 2017), besides other risk behaviours such as smoking or other illegal drugs consumption (Luthar & Becker, 2002; Luthar & D'Avanzo, 1999). This may be because adolescents from more affluent families have more disposable money of their own with which they buy substances such as alcohol. In fact, pocket money has been demonstrated to be a risk in previous research (Bellis et al., 2007; Lintonen, Rimpela, Vikat & Rimpela, 2000; Obradors-Rial, Ariza, Rajmil and Muntaner, 2018). Alternatively, studies have suggested that other factors such as excessive pressure to achieve and isolation from parents (literal and emotional) might make high affluence adolescents more vulnerable to substance use (Luthar & Latendresse, 2005).

In contrast to material affluence, perceived family wealth showed no association with lifetime and weekly alcohol consumption, but adolescents who perceived their families to be poor tended to report a higher frequency of lifetime drunkenness. It should be highlighted that these results were independent of family affluence which supports the finding that the correlation between them is low (Moor et al., 2019) and that the two socioeconomic indicators assess different aspects and should not be interchangeable (Hartley, Levin & Currie, 2016; Koivusilta, Rimpela & Kautiainen, 2006). Moreover, previous researchers have found that the perception of the subjective socioeconomic status affects wellbeing through psychosocial mechanisms related to anxiety and stress derived from a perception of a low living standard in comparisons with others (Kawachi, Kennedy and Glass, 1999; Wilkinson & Pickett, 2006). Irrespective of the level of material assets within the household, our findings expand the growing evidence that alcohol consumption, as with other stress-related behaviours, are more common among individuals who perceive themselves as disadvantaged compared to others (Elgar, Canale, Wohl, Lenzi & Vieno, 2018). In such cases, alcohol may be used as a coping strategy to manage stress and getting drunk at this age may help adolescents to attain a level of social status among their peers.

Regarding trends, the magnitude of social inequality in lifetime and weekly alcohol consumption did not change across survey years, however the effect of family affluence and perceived family wealth on lifetime drunkenness increased. Therefore, despite the overall downward trend in adolescent alcohol consumption, attention should be paid to persisting or even increasing social inequalities in alcohol consumption across years in order to target sub-groups of adolescents who remain particularly vulnerable to the negative effects of alcohol consumption. These findings are congruent with Liu et al. (2018). At international level, previous studies examining trends in socioeconomic inequalities in adolescent health did not include alcohol measures (Elgar et al., 2015).

Finally, the present study findings confirm the results of previous studies in Australia (Livingston, 2014), Germany (Richter, Kuntsche, de Looze and Pfoertner, 2013), and USA (Twenge & Park, 2017) which found that more stringent alcohol policies contributed to reducing socioeconomic inequalities in alcohol consumption. Having combined policies addressing alcohol availability and advertising were found to reduce the effect of family affluence on lifetime and weekly alcohol consumption (i.e. reducing the frequency of drinking relatively more in higher socioeconomic status groups which are characterized by higher levels of consumption). In addition, having a combination of policies was also found to reduce perceived family wealth inequalities in drunkenness as well as to mitigate the increasing effect of perceived family wealth across survey years in lifetime drunkenness. This suggests that stricter alcohol control and regulation might not only reduce the frequency of drunkenness relatively more in groups who perceive themselves as disadvantaged, but also reduce the differences in drunkenness between groups across years.

The present study has some limitations. Firstly, data were self-reported which may lead to underestimation due to biases such as non-response, under-reporting, recall, and social desirability. However, all data sources included in this study followed rigorous international protocols to ensure optimal validity and comparability and to minimize potential sources of bias (Roberts et al., 2007). Another important caveat is the failure to incorporate enforcement of policies into the regression analysis. Unfortunately, such information was not systematically available for the complete international sample. Future studies should also examine possible different effects of alcohol legislation according to other sociodemographic variables such as sex or age, as other studies have found for tobacco control policies (Pförtner et al., 2015) and marijuana laws (Hasin et al., 2015).

Nevertheless, this study has some major strengths. First, the large sample size representative for 33 countries/regions in Europe and North America across a 12-year period. Second, a comprehensive set of alcohol control policies and three different adolescent alcohol measures have been considered in the analyses. Finally, to the best of our knowledge, this is the first international study to examine the association of alcohol control policies with social inequality (measured by family affluence and perceived family wealth) and with trends in social inequality (for each socioeconomic indicator separately) in adolescent alcohol drinking outcomes.

## Conclusion

This study provides an overview of the relationship between national alcohol control policies, material and perceived socioeconomic inequalities, and alcohol consumption in adolescents over a 12-year period. Generally, single policy measures seem to have no or only limited effect, but a combination of policies seems to be more effective in reducing adolescent drinking. Alcohol pricing policy appeared to be the most successful single measure, which should be taken into account in discussions on alcohol taxation and minimum price per unit. Although socioeconomic inequalities in adolescent alcohol consumption have persisted and even increased across survey years, this study showed that combined alcohol control policy can help in reducing them.

## Declaration of Competing Interests

None

## References

[bib0001] Amphora Project. (2010). Country reports on alcohol policy.

[bib0002] Babones S.J. (2009). Social inequality and public health.

[bib0003] Bellis M.A., Hughes K., Morleo M., Tocque K., Hughes S., Allen T. (2007). Predictors of risky alcohol consumption in schoolchildren and their implications for preventing alcohol-related harm. Substance Abuse Treatment Prevention and Policy.

[bib0004] Brand D.A., Saisana M., Rynn L.A., Pennoni F., Lowenfels A.B. (2007). Comparative analysis of alcohol control policies in 30 countries. Plos Medicine.

[bib0005] Burton R., Henn C., Lavoie D., O'Connor R., Perkins C., Sweeney K.& . (2017). A rapid evidence review of the effectiveness and cost-effectiveness of alcohol control policies: An English perspective. Lancet (London, England).

[bib0006] Carragher N., Byrnes J., Doran C.M., Shakeshaft A. (2014). Developing an alcohol policy assessment toolkit: Application in the western Pacific. Bulletin of the World Health Organization.

[bib0007] CBS. (2016). Available from: http://www.cbs.gov.il/reader/cw_usr_view_Folder?ID=141.

[bib0008] Collins S.E. (2016). Associations between socioeconomic factors and alcohol outcomes. Alcohol Research-Current Reviews.

[bib0009] Currie C., Molcho M., Boyce W., Holstein B., Torsheim T., Richter M. (2008). Researching health inequalities in adolescents: The development of the Health Behaviour in School-Aged Children (HBSC) family affluence scale. Social Science & Medicine.

[bib0010] Donaldson G.W. (1998). Ridit scores for analysis and interpretation of ordinal pain data. European Journal of Pain-London.

[bib0011] Elgar F.J., McKinnon B., Torsheim T., Schnohr C.W., Mazur J., Cavallo F. (2016). Patterns of socioeconomic inequality in adolescent health differ according to the measure of socioeconomic position. Social indicators research.

[bib0012] Elgar F.J., Pförtner T., Moor I., de Clercq B., Stevens G., Currie C. (2015). Socioeconomic inequalities in adolescent health 2002–2010: A time-series analysis of 34 countries participating in the Health Behaviour in School-aged Children study. Lancet (London, England).

[bib0013] Elgar F.J., Canale N., Wohl M., Lenzi M., Vieno A. (2018). Relative deprivation and disordered gambling in youths. Journal of Epidemiology and Community Health.

[bib0014] European Centre for Monitoring Alcohol Marketing. (2016). Regulations on alcohol marketing.

[bib0015] Eurostat. Eurostat datbase (2016). Available from: http://ec.europa.eu/eurostat/data/database.

[bib0016] Feldstein-Ewing S., Sakhardande A., Blakemore S.J. (2014). The effect of alcohol consumption on the adolescent brain: A systematic review of MRI and fMRI studies of alcohol-using youth. Neuroimage-Clinical.

[bib0017] Gomes de Matos E., Kraus L., Hannemann T.-.V., Soellner R., Piontek D. (2017). Cross-cultural variation in the association between family's socioeconomic status and adolescent alcohol use. Drug and Alcohol Review.

[bib0018] Goodman E., Huang B., Schafer-Kalkhoff T., Adler N.E. (2007). Perceived socioeconomic status: A new type of identity that influences adolescents' self-rated health. Journal of Adolescent Health.

[bib0019] Hanson M.D., Chen E. (2007). Socioeconomic status and health behaviours in adolescence: A review of the literature. Journal of Behavioral Medicine.

[bib0020] Hartley J.E.K., Levin K., Currie C. (2016). A new version of the HBSC Family Affluence Scale - FAS III: Scottish qualitative findings from the international FAS development study. Child Indicators Research.

[bib0021] Hasin D.S., Wall M., Keyes K.M., Cerdá M., Schulenberg J., O'Malley P.M. (2015). Medical marijuana laws and adolescent marijuana use in the USA from 1991 to 2014: Results from annual, repeated cross-sectional surveys. The Lancet Psychiatry.

[bib0022] Holm A.L., Veerman L., Cobiac L., Ekholm O., Diderichsen F. (2014). Cost-effectiveness of preventive interventions to reduce alcohol consumption in Denmark. PloS one.

[bib0023] Inchley J., Currie D., Vieno A., Torsheim T., Ferreira-Borges C., Weber M.M. (2018). Adolescent alcohol-related behaviours: Trends and inequalities in the who European region, 2002–2014.

[bib0024] Jackson N., Denny S., Sheridan J., Fleming T., Clark T., Peiris-John R. (2017). Uneven reductions in high school students' alcohol use from 2007 to 2012 by age, sex, and socioeconomic strata. Substance Abuse.

[bib0025] Kawachi I., Kennedy B.P., Glass R. (1999). Social capital and self-rated health: A contextual analysis. American Journal of Public Health.

[bib0026] Koivusilta L.K., Rimpela A.H., Kautiainen S.M. (2006). Health inequality in adolescence. Does stratification occur by familial social background, family affluence, or personal social position?. BMC public health.

[bib0027] Kokkevi A., Stavrou M., Kanavou E., Fotiou A., Richardson C. (2018). Adolescents in Greece in time of economic crisis. Child Indicators Research.

[bib0028] Lavikainen H., Salmi V., Aaltonen M., Lintonen T. (2011). Alcohol-related harms and risk behaviours among adolescents: does drinking style matter. Journal of Substance Use.

[bib0029] Lintonen T., Rimpela M., Vikat A., Rimpela A. (2000). The effect of societal changes on drunkenness trends in early adolescence. Health Education Research.

[bib0030] Liu Y., Lintonen T., Tynjala J., Villberg J., Valimaa R., Ojala K. (2018). Socioeconomic differences in the use of alcohol and drunkenness in adolescents: Trends in the Health Behaviour in School-aged Children study in Finland 1990-2014. Scandinavian Journal of Public Health.

[bib0031] Livingston M. (2014). Trends in non-drinking among Australian adolescents. Addiction (England).

[bib0032] Luthar S.S., Latendresse S.J. (2005). Children of the affluent - Challenges to well-being. Current Directions in Psychological Science.

[bib0033] Luthar S.S., Becker B.E. (2002). Privileged but pressured? A study of affluent youth. Child Development.

[bib0034] Luthar S.S., D'Avanzo K. (1999). Contextual factors in substance use: A study of suburban and inner-city adolescents. Development and Psychopathology.

[bib0035] MAKStat. (2016). Available from: http://www.stat.gov.mk/Default_en.aspx.

[bib0036] Meier P.S., Holmes J., Angus C., Ally A.K., Meng Y., Brennan A. (2016). Estimated effects of different alcohol taxation and price policies on health inequalities: a mathematical modelling study. Plos Medicine.

[bib0037] Miech R.A., Johnston L.D., O'Malley P.M., Bachman J.G., Schulenberg J.E., Patrick M.E. (2018). Monitoring the future: National survey results on drug use, 1975–2017: Volume I, secondary school student.

[bib0038] Miron J.A., Tetelbaum E. (2009). Does the minimun legal drinking age save lives?. Economic Inquiry.

[bib0039] Moor I., Kuipers M.A.G., Lorant V., Pfoertner T.-.K., Kinnunen J.M., Rathmann K. (2019). Inequalities in adolescent self-rated health and smoking in Europe: Comparing different indicators of socioeconomic status. Journal of Epidemiology and Community Health.

[bib0040] Moore T.G., McDonald M., Carlon L., O'Rourke K. (2015). Early childhood development and the social determinants of health inequities. Health Promotion International.

[bib0041] Moreno-Maldonado C., Moreno C., Ramos P., Rivera F. (2018). Measuring the socioeconomic position of adolescents: A proposal for a composite index. Social Indicators Research.

[bib0042] Mulder J., de Greeff J. (2013). Eyes on Age - A research on alcohol age limit policies in European member states. Legislation, enforcement and research.Utrech.

[bib0043] Nelson J.P. (2015). Binge drinking and alcohol prices: A systematic review of age-related results from econometric studies, natural experiments and field studies. Health Economics Review.

[bib0044] Newbury-Birch D., Walker J., Avery L., Beyer F., Brown N., Jackson K. (2009). Impact of alcohol consumption on young people: A systematic review of published reviews.

[bib0045] Obradors-Rial N., Ariza C., Rajmil L., Muntaner C. (2018). Socioeconomic position and occupational social class and their association with risky alcohol consumption among adolescents. International Journal of Public Health.

[bib0046] Pförtner T.K., Moor I., Rathmann K., Hublet A., Molcho M., Kunst A.E., Richter M. (2015). The association between family affluence and smoking among 15-year-old adolescents in 33 European countries, Israel and Canada: The role of national wealth. Addiction (England).

[bib0047] Plunk A.D., Cavazaos-Rehg P., Bierut L.J., Grucza R.A. (2013). The persistent effects of minimum legal drinking age laws on drinking patterns later in life. Alcoholism-Clinical and Experimental Research.

[bib0048] Rabinovich L., Brutscher P.B., de Vries H., Tiessen J., Clift J., Reding A. (2009). The affordability of alcoholic beverages in the European union.

[bib0049] Richter M., Kuntsche E., de Looze M., Pfoertner T.-.K. (2013). Trends in socioeconomic inequalities in adolescent alcohol use in Germany between 1994 and 2006. International Journal of Public Health.

[bib0050] Roberts C., Currie C., Samdal O., Currie D., Smith R., Maes L. (2007). Measuring the health and health behaviours of adolescents through cross-national survey research: Recent developments in the Health Behaviour in School-aged Children (HBSC) study. Journal of Public Health.

[bib0051] Rosstat. (2016). Available from: http://www.gks.ru/wps/wcm/connect/rosstat_main/rosstat/en/main.

[bib0052] Siegfried N., Pienaar D.C., Ataguba J.E., Volmink J., Kredo T., Jere M. (2014). Restricting or banning alcohol advertising to reduce alcohol consumption in adults and adolescents. Cochrane Database of Systematic Reviews.

[bib0053] Snijders T., Bosker R.J. (2012). Multilevel analysis : An introduction to basic and advanced multilevel modelling.

[bib0054] Statcan. (2016). Available from: http://www.statcan.gc.ca/eng/start.

[bib0055] Subbaraman M.S., Kerr W.C. (2013). State panel estimates of the effects of the minimum legal drinking age on alcohol consumption for 1950 to 2002. Alcoholism-Clinical and Experimental Research.

[bib0056] Twenge J.M., Park H. (2017). The decline in adult activities among U.S. Adolescents, 1976-2016. Child Development.

[bib0057] Ukrstat. (2016). Available from: https://ukrstat.org/en/operativ/oper_new_e.html.

[bib0058] Wagenaar A.C., Salois M.J., Komro K.A. (2009). Effects of beverage alcohol price and tax levels on drinking: A meta-analysis of 1003 estimates from 112 studies. Addiction (England).

[bib0059] Wagenaar A.C., Toomey T.L. (2002). Effects of minimum drinking age laws: Review and analyses of the literature from 1960 to 2000. Journal of Studies on Alcohol.

[bib0060] Waller R., Murray L., Shaw D.S., Forbes E.E., Hyde L.W. (2018). Accelerated alcohol use across adolescence predicts early adult symptoms of alcohol use disorder via reward-related neural function. Psychological Medicine.

[bib0061] Wilkinson R.G., Pickett K.E. (2006). Income inequality and population health: A review and explanation of the evidence. Social Science & Medicine.

[bib0062] World Health Organization. (2016a). Alcohol policy timeline database.

[bib0063] World Health Organization. (2016b). Global information system on alcohol and health.

[bib0064] World Health Organization. (2018). Global status report on alcohol and health 2018. Available from:https://www.who.int/substance_abuse/publications/global_alcohol_report/gsr_2018/en/.

[bib0065] Zaborskis A., Sumskas L., Maser M., Pudule I. (2006). Trends in drinking habits among adolescents in the Baltic countries over the period of transition: HBSC survey results, 1993-2002. BMC Public Health.

[bib0066] Zhang N., Caine E. (2011). Alcohol policy, social context, and infant health: The impact of minimum legal drinking age. International Journal of Environmental Research and Public Health.

